# Women Empowerment through Health Information Seeking: A Qualitative Study

**Published:** 2015-04

**Authors:** Alireza Nikbakht Nasrabadi, Sakineh Sabzevari, Tayebeh Negahban Bonabi

**Affiliations:** 1 Department of Medical Surgical Nursing, Nursing and Midwifery College, Tehran University of Medical Sciences. Tehran, Iran;; 2 Department of Medical Surgical Nursing, Nursing and Midwifery College, Kerman University of Medical Sciences, Kerman, Iran;; 3 Department of Community Health Nursing, Nursing and Midwifery College, Kerman University of Medical Sciences, Kerman, Iran

**Keywords:** Women, Information Seeking, Empowerment, Health Information

## Abstract

**Background:**

Today, women empowering is an important issue.  Several methods have been introduced to empower women. Health information seeking is one of the most important activities in this regard. A wide range of capabilities have been reported as outcomes of health information seeking in several studies. As health information seeking is developed within personal-social interactions and also the health system context, it seems that the qualitative paradigm is appropriate to use in studies in this regard. This study aimed to explore how women’s empowerment through health information seeking is done.

**Methods:**

In this qualitative content analysis study, data collection was done with regard to inclusion criteria, through purposive sampling by semi-structured interviews with 17 women and using documentation and field notes until data saturation. Qualitative data analysis was done constantly and simultaneous with data collection.

**Results:**

Four central themes were emerged to explain women’s empowerment through health information seeking that included: a) Health concerns management with three subcategories of Better coping, Stress management, Control of situation, b) Collaborative care with two subcategories of Effective interaction with health professions and Participation in health decision making c) Individual development d) Self-protection with four sub- categories of Life style modification,  Preventive behaviors promoting, Self-care promoting, and  medication adherence.

**Conclusion:**

The results of this study indicate the importance of women empowerment through foraging their health information seeking rights and comprehensive health information management.

## Introduction

Empowering women is an important subject of today’s world. The term “empowered” refers to the possession of legal power or autonomy to act.^1^ Empowerment is a process through which individuals, societies, and organizations gain the control of their important matters.^2^ The aim of moving toward empowerment is to acquire the necessary activities in order to prevent the threats and improve the positive aspects of life.^3^ Acquisition of such power is based on the knowledge and skills and promotes the quality of life. Moreover, it facilitates the ability to choose appropriate strategies for controlling the resources required for reaching favorable consequences.^4^

Previous studies showed that empowerment of patients resulted in favorable health outcomes,^5^ such as increased power of decision-making, freedom for making choices and accepting the responsibility,^6^ developed trust in relations, informed choice,^7^ facilitation of adaptation and well-being, hopefulness, increased speed of personal development, awareness of one’s own world, identification of one’s own strengths and abilities, feeling more powerful, higher self-confidence, higher personal satisfaction, higher self-efficacy, and eventually, and improved quality of life.^8^

Based on available reports, there are various methods for empowering women, and the most important methods include the economic independence,^9^ enhancement of skills for information technology and communication^10^ and promotion of health knowledge.^11^ Health information seeking behaviors can promote health knowledge and, consequently, formulate the judgments, beliefs, and attitudes toward healthy behaviors and, eventually, acquisition of adequate knowledge for identifying the alternatives and available resources for doing different activities and taking into account the positive and negative aspects of issues.^12^ In this respect, it is necessary to empower patients toward acquiring, processing, and understanding the basic health information.^13^

Regarding the key role of women in improvement of lifestyle and, consequently, the community health, the promotion of women’s health information is a fundamental strategy for empowering them.^14^ The available literature in this regard mentioned various capabilities as the outcomes of seeking health information. These capabilities include the contribution to medical decision-making,^15^ better adaptation, lower levels of stress, receiving higher social support, and reaching favorable changes in lifestyle.^16^ However, few studies have been performed on seeking health information in general and capabilities acquired following health information seeking behaviors in particular in Iran. The available reports in this regard are limited only to the needs, motivations, and barriers to seeking health information of patients with heart diseases.^17^

Considering that the process of seeking health information is developed within personal-social interactions and also the health system, it seems appropriate to use the qualitative paradigm in studies in this regard. As different studies have introduced women as more active seekers compared to men,^18^ this study focused on women.

This study was conducted to discover women’s empowerment through seeking health information in order to assist healthcare providers to identify and then plan for achieving favorable empowerments related to health.  

## Materials and Methods

This study was conducted with a qualitative approach, using conventional content analysis. Qualitative content analysis is an appropriate method to obtain variable and reliable results from textual data. This method is used to create new knowledge and ideas, and provide facts and guide lines, aiming to condense a broadly described phenomenon, with achievements of concepts or descriptive classes of the phenomenon.^19^

The present study is a part of a large study with grounded theory. The participants were 17 women through purposeful selection from rural and urban health centers, organizations associated with women’s affairs, parks, and offices of the Rafsenjan city in Iran. The study inclusion criteria were middle-aged married Iranian women, with the ability to communicate well desire to take part in study, and with favorable physical, mental and cognitive conditions for sharing their experiences. Generally, the participants were selected from a diverse range in terms of age, education, occupation, place of residence (rural, urban), health status, etc. For purposive maximum variation sampling, the researchers made an attempt to interview with the knowledgeable women who could provide broad insight regarding research question, through friends, colleagues and health care providers.

Following approval of the project by the Research Deputy of Kerman University of Medical Sciences No 93/10/60/24158, semi-structured interviews were conducted by a research team member, familiar with interviewing techniques. Sampling continued until saturation of data. Saturation was diagnosed by not emerging new categories and until emerged categories were enriched. All participants who were invited for the interview accepted the invitation, and continued their participation until the end of study. After agreement on time and place between the interviewer and interviewees, and obtaining consent of eligible participants, in an intimate and relaxing setting, before the interview, the researcher explained the study objectives and reasons for their selection for interview and participation, and clarified benefits of the study for them and the nurses, and how they could access final results. Then, according to the study objectives, the subject of research questions, and the approach adopted, the researcher posed several possible questions according to the interview guide such as: “would you like to tell me about your health information seeking experiences?”, “In what way do you think health information seeking make difference in your every day routines?”, and “what has been changed due to your health information?” To correctly direct the research, and according to previous interviews, the interview guide was changed after each interview which lasted between 45 minutes and 120 minutes. Interviews were conducted at home or workplace of either the researcher (School of Nursing and Midwifery-Rafsanjan) or participants. Interviews were transcribed immediately after completion. To that end, recorded interviews were listened to several times, and then typed verbatim in Microsoft Word, which led to researcher’s immersion in data. Along with interviews, the participants’ states and characteristics were noted. Furthermore, the researcher attended Rafsanjan’s public library, and reviewed 10 copies of local and national newspapers, family magazines, and columns on medical topics, and used them as data. Contents of these topics focused mostly on women’s questions and answers about health issues. The researcher also attended health centers and studied educational booklets, pamphlets, CDs, brochures, and used them in analysis of data.

Data were analyzed with an inductive approach by research team, using constant comparative method in stages: transcribing recorded interviews in Microsoft Word® software, and determining meaning units, which included women’s statements in interviews and materials obtained from observations, documentations, and field notes on women’s empowerment through health information seeking, coding and labeling meaning units, review of codes with interview text and information obtained from other sources, revision and comparison of codes in terms of similarities and differences, merging similar codes and categorization, development of categories according to similarity and suitability, revision and comparison of categories according to data to ensure rigor of codes, and finally, identification of themes associated with women’s empowerment through health information seeking.^20^

To ensure accuracy and reliability of qualitative data, standards of scientific rigor were applied including credibility, dependability, transferability, and confirm ability, as proposed by Lincoln and Guba.^21^ To ensure credibility of data, prolonged involvement (over a year), triangulation in data collection (field notes, transcriptions, interviews and documentations), peer check and constant comparison were used. Member check was used to assess dependability of data (to ensure stability and reliability of data). To this end, comments from colleagues familiar with qualitative approach and review of participants’ transcriptions were used. These and field notes were also presented to two nursing professors to confirm the results. Transferability was provided by rich description of data.

*Ethical Considerations*

This study was conducted after approval of Ethics Committee of Kerman University of Medical Sciences, code No 93/133. Principles of ethics in research including informed consent, anonymity, confidentiality, and participants’ rights to withdraw from study were observed. Moreover, study objectives, confidentiality of data, and recording of interviews were explained to the participants prior to interviews, and their verbal consents were obtained. 

## Results

A total of 17 married women, aged 25 to 60 years participated in this study; of them, 5 were housewives, 10 employees, and 2 self-employed. Six women lived in villages and the rest were city dwellers. Six participants had known diseases and were receiving treatment, and the rest appeared healthy. Two interviews were held in participants’ private homes, one in researcher’s home, two at participants’ workplace, and twelve researcher’s office at Rafsenjan School of Nursing and Midwifery. Table 1 presents characteristics of participating women.

**Table 1:** Characteristics of participants in the study

**Participant**	**Age**	**Education**	**Health status**	**Occupation**	**Place of residence**	**Interview location**
P1	50	Illiterate	Patient	Housewife	City	Participant home
P2	47	Master’s degree	Healthy	Employee	City	Researcher work Place
P3	40	Bachelor’s degree	Healthy	Employee	City	Participant home
P4	25	Diploma	Healthy	Housewife	City	Researcher work Place
P5	50	Diploma	Healthy	Employee	City	Researcher work Place
P6	40	Associate degree	Healthy	Employee	Village	Researcher home
P7	30	Diploma	Healthy	Housewife	Village	Researcher work Place
P8	60	Seminary	Patient	Housewife	Village	Researcher work Place
P9	51	Bachelor’s degree	Patient	Employee	City	Researcher work Place
P10	55	Diploma	Patient	Self employed	City	Researcher work Place
P11	35	Bachelor’s degree	Healthy	Employee	City	Researcher work Place
P12	37	Seminary	Patient	Housewife	Village	Researcher work Place
P13	50	Associate degree	Healthy	Employee	City	Researcher work Place
P14	38	Bachelor’s degree	Healthy	Employee	City	Researcher work Place
P15	52	Bachelor’s degree	Patient	Self employed	Village	Researcher work Place
P16	51	Diploma	Healthy	Employee	City	Participant work Place
P17	35	Master’s degree	Healthy	Employee	Village	Participant work Place

Four central themes were emerged to explain women’s empowerment through health information seeking that includes: a) Health concerns management with three subcategories of Better coping, Stress management, Control of situation b) Collaborative care with two subcategories of Effective interaction with health professions and Participation in health decision making c) Individual development d) Self-protection with four sub- categories of Life style modification,  Preventive behaviors promoting, Self-care promoting, and medication adherence.

1. Management of health concerns
  The theme of management of health concerns was seen in experiences of most women in different ways. It comprised the following subthemes: adaptation with health problems, stress management, and control of the situation.
  1.1. Adaptation with health problems
    Based on the data analysis, acquisition of health information was accompanied with the increase in women’s adaptability and facilitation of accepting the problems caused by the disease, especially in patients with chronic diseases. For instance, participant 11 explained her experiences of adapting with her disease as, “I was easily persuaded with specialists’ explanation about the subjects that were ambiguous for me and could better cope with my disease and accept it.” Another woman described her experiences of how health information supported her to cope with the symptoms of the disease as follows, “The information helped me accept my disease. I mean I found the disease can’t wear me down. I try to do all my tasks so my life doesn’t lag.” (Participant no. 12)
    The better interaction with the disease was also one of the factors facilitating the adaptation in some women. In this regard, participant 15 stated, “When I received the information about hypertension, I learnt to change my diet. I don’t eat salt and fat. I feel I can better interact with my disease that is always with me.”
    Moreover, women stated that they experienced higher tolerance with the course of the disease and easier adaptation with symptoms of the disease after acquisition of adequate health information. In this regard, participant 7 explained, “If I get enough information, I can better cope with the disease, and my patience for completing my treatment increases because I’m in high spirit.”
    Being optimistic about the prognosis of the disease, being hopeful for having better life, being assured to treat symptoms of the disease, and eventually, reaching higher levels of satisfaction were other capabilities of women. Participant 11 explained about the contribution of health information to her optimism about the prognosis of her chronic disease as follows, “The information I had before and also the information I got in the seminar of the Center for Special Diseases helped me accept that really no movement disorder is supposed to happen for me, and I can achieve the relative recovery.” (Participant 11)
    On the hopefulness arising from getting information, another participant pointed out, “If I had a disease but knew it adequately, I think I would be more hopeful about life.” (Participant 7)
  1.2. Stress management
    Most of the women had experienced the stress and concerns caused by lack of information and mentioned the inhibition of internal fear, reduction of anxiety and stress, and consequently, feeling of peace and satisfaction following the acquisition of health information. For instance, participant 5 explained her experience as follows, “I was afraid of labor; but I tried to overcome my fear and have a good labor by getting information.”
    The women introduced the feeling of internal satisfaction as the general consequence of stress management. In this regard, participant 9 expressed, “Because I could overcome my fear, I internally feel very well. I have gained high self-confidence.”
  1.3. Control of the situation
    The participating women had experienced the ambiguous conditions, failure to understand the situation, and inability to predict the conditions ahead very apprehensively, stressfully, and fearfully. They introduced the acquisition of health information as a way of understanding the situation and feeling powerful to control the situation, predict future events, and finally, try to prevent and control the occurrence of unwanted complications. In this regard, one of the women described her experience as follows, “Fingers of my son had been cut; I didn’t have any information about wounds, tendons, and amputations. If I had known, at least I could have understood what would happen then. I wondered what would happen then. Could it be grafted? Could it be OK? Finally, the surgeon explained about the surgery and healing of grafts and made us control the condition to some extent, and we could do something to take my son to the hospital” (Participant 2).
2. Participatory care
  According to the data analysis, another important skill gained by women following the acquisition of health information was participation in caring for themselves, family members, or others. This theme consisted of two subthemes: effective interaction with health personnel and participation in medical decision-making.
  2.1. Effective interaction with health personnel
    The women stated that they had experienced the ability to establish verbal communication with health professions and familiarity with the medical common language and the resulting facilitation of verbal communication after getting health information. For instance, a midwife participant expressed her experiences of the role of patients’ health information in understanding health instructions as follows:“An example is the manner to apply disinfectants at episiotomy site after labor. If a woman has enough information in this regard, I see that she understands us in any way we explain to her, and she heeds well and doesn’t get infected. But, patients coming with an infection at episiotomy site say that they weren’t instructed or were instructed in a way that they didn’t understand it at all.” (Participant 11)
    Furthermore, the women mentioned enough health information as a means of trusting healthcare providers and the consequent facilitation of receiving instructional materials, spending less time for interacting with healthcare providers, and enjoying the process of treatment. Participant 15 stated her distrust arising from having no health information as follows, “Many years ago, my doctor prescribed me a pill in the last month of my pregnancy. I didn’t have any information, and when I saw he prescribed me a pill, I wondered, ‘oh I’m pregnant, I shouldn’t use the drug. Maybe, he doesn’t know.’ I didn’t use those pills. But later, I realized they were iron pills and necessary for me.”
    Some women found the increased support of health personnel and success in acquisition of complementary and detailed information from health personnel as other outcomes of having health information. In this regard, participant 9 pointed out, “When I already have information, I’ll ask the doctor more accurate questions. Maybe, when the doctor sees that I know something, he explains more and fully to me.”
  2.2. Participation in medical decision-making
    According to the participants, another outcome of seeking health information was the capability to participate in healthcare. In this regard, one of the women stated, “I kind of cooperated with the doctor for treatment of my child. For example, when a doctor gave an antibiotic for my child’s asthma, but I thought not to give him the antibiotic, and I recognized that he was exposed to allergens and his disease has relapsed, I took him to another doctor. I told the doctor that I thought I shouldn’t give him the antibiotic, and the doctor confirmed that I did the right thing and that I didn’t need to give him the antibiotic.” (Participant 3)
    On the participation in healthcare, another participant explained, “When I know enough about the causes and treatment of the disease, I think I can deal with the disease better, and certainly I will be of better help for the person who wants to treat me.” (Participant 9)
3. Personal development
  According to the women’s experiences, health information seeking behaviors resulted in further familiarity with different sources of health information, improvement of physical and mental abilities, higher self-confidence, and an increase in participation in social activities. As to the familiarity with different sources of health information, participant 7 explained, “It repeatedly occurred that I had some information, but when I discussed them somewhere, I found they were wrong. Then, I was directed to some sites, books, newspapers, or other media, and I could get more complete information.”
  Furthermore, the women introduced their ability to differentiate the proper health information from improper information, better justify health-related issues, and guide others as other outcomes of seeking health information. For instance, participant 9 stated, “I’m satisfied with what happened to my well-being. It’s changed my life. It’s good both physically and mentally. I’m in touch with the society. I try to impact the people I’m in touch with. My mother’s old, but I think she gets better when I talk with her. It’s also true about my family, such as my sister or my sister-in-law.” (Participant 9)
  According to the participants, other capabilities included feeling powerful to deal with health difficulties, trying to minimize the limitations, managing the health difficulties successfully, having easier life, and being encouraged to acquire more accurate health information. On being encouraged to acquire more health information, participant 17 pointed out, “Well, when I find my information has increased, I feel happy. I’ll be encouraged to follow up and seek more information if I have another problem.”
4. Self-protection
  Self-protection was another theme for describing the empowerment of women through seeking health information. This theme consisted of the subthemes of the lifestyle modification, promotion of preventive behaviors, self-care promotion, and enhancing adherence to treatment.
  4.1. Lifestyle modification
    The acquisition of health information had made the women modify their lifestyle and heed safe behaviors, such as healthy eating style, exercising, and being careful when purchasing food products. In this regard, one of the participants explained, “When I receive health information, I’ll be more sensitive about my health, my child’s, my husband’s, and others’ health and try to furnish them with health. I try to deal with health risk factors or prevent them.” (Participant 8)
  4.2. Promotion of preventive behaviors
    Results of the data analysis revealed that having received health information, the women took steps toward observing the behaviors that prevent diseases, including cancers and other chronic diseases, such as cardiovascular problems and diabetes, and also avoiding the disability. For instance, participant 10 explained, “Having information makes me compare my status with that information. For example, I provide myself with conditions possible for me to prevent the incidence of difficulties and diseases.”
    The sensitivity to the primary symptoms of diseases and performing the screening tests were other skills gained by women after seeking health information. On the use of information relevant to the analysis of the primary symptoms of breast cancer, participant10 stated, “For example, I use the information I learnt for early diagnosis of breast cancer. For example, I do the breast self-examination every month after the menstruation.”
    On the avoidance of disability, participant 15 expressed, “I’m concerned not to get sick; when you don’t get sick, you don’t pay any costs, and permanent disabilities that break the family and incur pains and deprive you of many things don’t occur.”
  4.3. Self-care promotion
    The participating women had gained the capability to do self-care while looking for health information. In this regard, participant9 pointed out, “When I suffered low back pain, I studied a lot. The disease, by and large, changed the path of my life and changed my mentality. It made me pay more attention to myself, spend more time for myself, and care for myself more than before.”
    Similar to the self-care, caring for the family was another field empowering the women through making them seek health information. In this regard, participant 17 stated, “I get information about things that are useful for my child’s well-being and use it in caring for my child.”
  4.4. Improvement of the adherence to treatment
    Another capability introduced by the women was their optimism for the prognosis of diseases, completion of the entire treatment process, better adherence to diet, and acceptance of and accurate adherence to the prescribed medication. On the accurate adherence to medication after getting health information, participant 17 explained, “I think having information made me adhere to the treatment better. For example, when I was acquiring information about symptoms and consequences of hypothyroidism, I used my drugs more carefully. But, if I didn’t have any information, I’d doubt the effectiveness of the drug, so I wouldn’t use them correctly. The information helped me a lot not to miss my drugs and emphasize using them on time.” Another participant suffering a chronic disease expressed, “The information I got made me optimistic about my disease and helped me follow my doctor’s advice. The shaking and disability of my hands really decreased within the first two months, and numbness of my lower limbs finished. I see that I feel more powerful.” (Participant 11)

## Discussion

The results of this study presented a new broad insight toward the areas and manner of empowering women through health information seeking behaviors. The participants experienced the empowerment through adapting with health problems more favorably, managing the stress, and controlling the situation. The available reports showed contradictory results in this regard. On the one hand, some studies argued that seeking health information was an important factor for promotion of people’s adaptability, as it assisted people to understand the threats to health and their accompanying challenges^22^ and guided people to manage the threatening situation, know the stressors, and consequently, deal with the stressors more powerfully.^23^ On the other hand, results of some other studies showed that people were adapted through refraining from receiving the information related to the health-threatening situations. These situations were mentioned in relation to the cancer information in general and genetic screening for examining the probable incidence of cancer in particular.^24^ The contradiction seemed to be associated with the conditions of seekers and the type of information they sought. In this respect, people sometimes adapt with the stress and control it through refraining from receiving the information on the imminence or incurability of a disease, the condition that is out of their control; on the contrary, people sometimes would reach the inner satisfaction and adaptation through seeking information that empower them and enhance their feeling of controlling the situation.

In this study, seeking health information through facilitation of effective interactions with health personnel and participation in medical decision-making empowered the women to participate in caring for themselves. Results of some studies revealed that the patients were empowered through seeking health information for communicating with health personnel^14^ and asking questions effectively, understanding the information more easily, and reducing the number of unnecessary visits.^25^ A study conducted in the United States showed that 92% of patients with cancer believed that health information empowered them to have more useful conversations with the doctor and medical decision making.^26^ Moreover, some health personnel with limited skills and information react defensively against patients who have information and lead the patients toward their desired choices through explaining their own experimental ideas within short conversations.^27^ These results might reveal gaps in reaching a participatory health system that recognizes the responsibility of patients for acquiring information and making medical decisions.

The results of this study showed that seeking health information empowered women to promote their self-confidence, filter the information, manage life problems successfully, feel strong against health problems and disabilities, and be encouraged to seek more information. The above results conformed to those in the literature. In this respect, the acquisition of health information improved the role of patients in managing their self-care^28^ and empowered them to filter the information they received.^29^ Moreover, a significant correlation was reported between communication of health information and higher self-confidence and also being encouraged to seek more information.^30^ These results highlighted the empowerment of patients through seeking health information.

The women participating in this study were eventually empowered through modifying their lifestyle, promoting their preventive behaviors, improving their adherence to treatment, and promoting self-care toward the self-protection. Results of a study indicated that seeking information on cancer significantly correlated with level of heeding the cancer-preventing lifestyle.^31^ Furthermore, another study showed the predictive role of receiving routine health information in occurrence of preventive behaviors, such as the number of days of exercising per week, daily intake of fruit and vegetables, and doing mammography.^32^

In a study, among the patients with HIV, those patients who had more health information seeking behaviors significantly adhered to the treatment more than others even after control of confounding variables.^33^ However, the acquisition of information referring to high-cost treatments^34^ and also contradictory health information had negative correlation with patients’ adherence to treatments.^35^ Moreover, the patients using multiple medications sought information from different sources, such as the media, friends, family, brochures, and magazines and did not adhere to treatments.^36^ It seems that the multiple medication regimen worries patients, especially when the medications are always changed. During the medical consultation with health professionals, if patients do not come to trust, they decide to collect the information from other sources and then decide about how to adhere to medication based on their perception. Moreover, as most patients receiving multiple medications are old and have cognitive disorders, they perceive the medical information in a different way, and consequently, the information may have adverse effects on their drug adherence. Therefore, these results emphasized the necessity for heeding the principles of effective communication of healthcare professions with such patients and receiving the feedback in medical consultation.

Similar to the results of this study, the findings of other studies showed that acquisition of health information was accompanied with the appearance of self-care behaviors, such as controlling side effects of drugs and symptoms of diseases, observing the diet, controlling complications of the diseases, and so forth.^37^

The findings represent our interpretation of the voice of women who had lifestyle change through health information seeking. This study was conducted for the first time in Iran and the results revealed the empowerment of women by health information seeking. And it seems that using triangulation strategy in data gathering and data analysis and also in-depth interviews and prolonged contact of researchers with participants produced reliable data and led to extracting valid results. Although researchers’ attempt was to ensure accuracy and reliability of qualitative data, it should be noted that this study had all qualitative research limitations in generalization of results. Thus it is necessary to repeat the study in different groups and cultural backgrounds. The results of this study highlighted the importance of empowering women through supporting their right to seek health information and manage health information comprehensively. 

## Conclusion

Undoubtedly, empowerment of women through acquiring health information increases their participation in healthcare and consequently, their involvement and sense of responsibility in prevention, protection and promotion of the health of themselves, their family, and the society, and facilitates the achievement of favorable health outcomes. 
